# Expression of Formate-Tetrahydrofolate Ligase Did Not Improve Growth but Interferes With Nitrogen and Carbon Metabolism of *Synechocystis* sp. PCC 6803

**DOI:** 10.3389/fmicb.2020.01650

**Published:** 2020-07-14

**Authors:** Shanshan Song, Stefan Timm, Steffen N. Lindner, Viktoria Reimann, Wolfgang R. Hess, Martin Hagemann, Eva-Maria Brouwer

**Affiliations:** ^1^Plant Physiology Department, Institute of Biosciences, University of Rostock, Rostock, Germany; ^2^Max Planck Institute of Molekular Plant Physiology, Potsdam-Golm, Germany; ^3^Faculty of Biology, Genetics and Experimental Bioinformatics, University of Freiburg, Freiburg im Breisgau, Germany

**Keywords:** C1 metabolism, cyanobacteria, carbon fixation, formate assimilation, metabolome, serine, transcriptomics

## Abstract

The introduction of alternative CO_2_-fixing pathways in photoautotrophic organism may improve the efficiency of biological carbon fixation such as minimizing the carbon loss due to photorespiration. Here, we analyzed the effects of creating a formate entry point into the primary metabolism of the cyanobacterium *Synechocystis* sp. PCC 6803. The formate-tetrahydrofolate ligase (FTL) from *Methylobacterium extorquens* AM1 was expressed in *Synechocystis* to enable formate assimilation and reducing the loss of fixed carbon in the photorespiratory pathway. Transgenic strains accumulated serine and 3-phosphoglycerate, and consumed more 2-phosphoglycolate and glycine, which seemed to reflect an efficient utilization of formate. However, labeling experiments showed that the serine accumulation was not due to the expected incorporation of formate. Subsequent DNA-microarray analysis revealed profound changes in transcript abundance due to *ftl* expression. Transcriptome changes were observed in relation to serine and glycine metabolism, C1-metabolism and particularly nitrogen assimilation. The data implied that *ftl* expression interfered with the signaling the carbon/nitrogen ratio in *Synechocystis*. Our results indicate that the expression of new enzymes could have a severe impact on the cellular regulatory network, which potentially hinders the establishment of newly designed pathways.

## Introduction

Inorganic carbon fixation by photoautotrophic organisms via the Calvin-Benson-Bassham (CBB) cycle represents the biochemical process that supplies organic carbon for almost all living organisms on Earth. In nature, factors limiting the growth of photosynthetic organisms vary among species and habitats and include the availability of water, light, and nutrients e.g., combined nitrogen sources (Evans, 1997). However, in modern agriculture, using fertilizers and often irrigation, CO_2_ fixation became the rate-limiting factor of crop plant yield under ambient conditions due to the inefficiency of the key CO_2_-fixing enzyme of the CBB cycle, the ribulose 1,5-bisphosphate carboxylase/oxygenase (RubisCO) (Long et al., 2006; Tcherkez et al., 2006; Bar-Even et al., 2010). RubisCO possesses a slow catalytic rate and an oxygenation side reaction producing the toxic byproduct 2-phosphoglycolate (2PG). 2PG must be metabolized through the photorespiratory pathway, which releases previously fixed CO_2_ and liberates NH_3_ (Bauwe et al., 2010). Many attempts were undertaken to enhance photosynthetic carbon fixation, like engineering RubisCO toward higher catalytic efficiency and specificity for CO_2_ (Whitney et al., 2011). Other attempts aimed at increasing the CO_2_ concentration in close vicinity to RubisCO, which has been naturally achieved during evolution of different CO_2_-concentrating mechanisms (CCMs) in cyanobacteria, algae and C4 plants (Leegood, 2002; Badger and Price, 2003; Giordano et al., 2005; Long et al., 2018). In recent years, it has also been tried to optimize photorespiration in plants by expressing different artificial bypass reactions to improve the recycling of 2PG (Kebeish et al., 2007; Hagemann and Bauwe, 2016; South et al., 2019). Another *in vitro* approach established a carbon-conserving photorespiration by converting glycolate via glycolyl-CoA and glycolaldehyde into CBB cycle intermediates (Trudeau et al., 2018).

As an alternative to the improvement of the CBB cycle and photorespiration, which are intimately linked to plant primary metabolism, the generation of entirely new synthetic CO_2_-fixing pathways has been proposed. Shih et al. (2014) generated a synthetic photorespiratory CO_2_-fixing bypass in cyanobacteria, which provided the basis for an alternative carbon fixation pathway in cyanobacteria, algae and plants. Schwander et al. (2016) were able to design and prove an *in vitro* CO_2_ fixing pathway, the CETCH [(CoA)/ethylmalonyl-CoA/hydroxybutyryl-CoA] cycle that involves 11 enzymatic steps. The direct assembly of this synthetic pathway in living organisms is challenging due to limited understanding of the complex interplay among the different enzymes used in this synthetic network. Furthermore, the interference of the synthetic networks with the complex metabolic and regulatory background of the host organism can lead to undesired side reactions and toxicity (Schwander et al., 2016).

**FIGURE 1:**
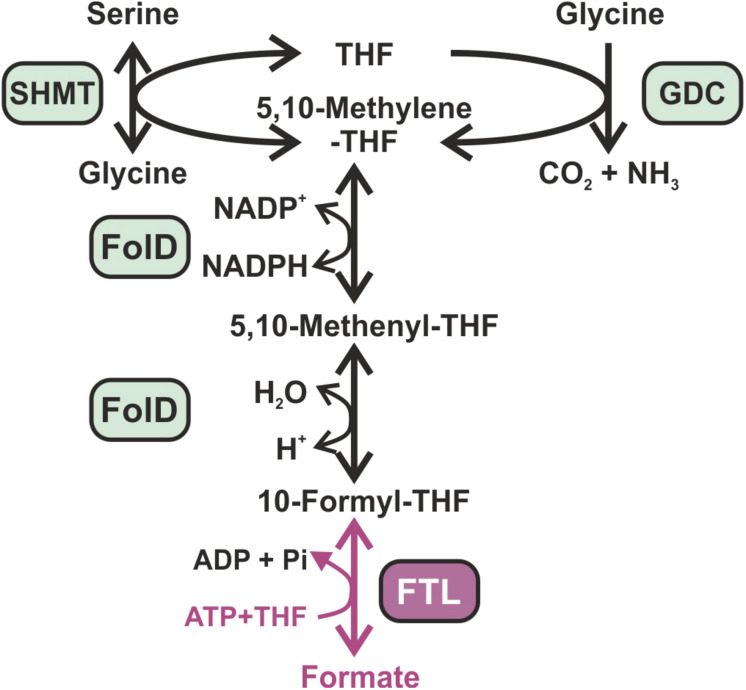
Synthetic formate assimilation (FA) pathway in *Synechocystis* sp. PCC 6803. Enzymes present in the *Synechocystis* host cell are marked in green, while the additional enzyme necessary for formate incorporation is marked in pink. FTL, formate-THF ligase; FolD, bifunctional methylene-THF dehydrogenase/methenyl-THF cyclohydrolase; GDC, glycine decarboxylase complex; SHMT, serine hydroxymethyltransferase.

Recently, formate has been proposed as an ideal feedstock for bio-economy, because it can be produced at relatively high efficiency from multiple available resources such as the electrochemical reduction of CO_2_ and oxidization of natural gas (Bar-Even et al., 2013). Furthermore, formate is soluble and of low toxicity. Many methylotrophic organisms can grow with formate as sole carbon source (Marx et al., 2003). The establishment of additional CO_2_ reduction into formate in photoautotrophic organisms such as crop plants was proposed to support CO_2_ fixation via the CBB cycle (Bar-Even, 2018). The most valuable entry point of formate into primary carbon metabolism is via conversion into 10-formyl-tetrahydrofolate (formyl-THF) by the formyl-THF ligase (FTL) (Bar-Even, 2016). FTL catalyzes an ATP-dependent kinase reaction that gives rise to the intermediate formyl-phosphate and the activated formyl-group is then transferred on THF to give formyl-THF (Mejillano et al., 1989). FTL does not directly generate a carbon–carbon bond but it activates formate, making it a good electrophile for downstream reactions with a nucleophilic carbon atom. FTL is the only known naturally occurring formate-fixing reaction that supports formatotrophic growth (Bar-Even, 2018). In most organisms, formyl-THF naturally participates in the synthesis of purines and also takes part in the formylation of initiator methionyl-tRNA^*Met*^ in bacteria, mitochondria and chloroplasts. It can also be converted to methylene-THF via the bi-functional methylene-THF dehydrogenase/methenyl-THF cyclohydrolase (FolD) (Hanson and Roje, 2001). Subsequently, methylene-THF can, together with glycine, serve for serine biosynthesis via the serine-hydroxymethyltransferase (SHMT), which represents an important step in the C1-metabolism of most organisms (Figure 1). In plants and other oxygenic phototrophs, the CO_2_-releasing step via glycine cleavage in the photorespiratory pathway produces high amounts of methylene-THF, which is then used by SHMT to synthesize serine on the expense of a second glycine molecule. It has been discussed that an increased pool of methylene-THF due to efficient formate incorporation could turn photorespiration into less CO_2_-releasing or even CO_2_-fixing, when the glycine-decarboxylase reaction is reversed Recently, the formate-assimilation pathway including a reversed glycine decarboxylase flux was successively established in *E. coli*, proving the afore calculated kinetical feasibility and functionality of the designed CO_2_-fixing shunt (Bar-Even et al., 2010; Yishai et al., 2017; Bang and Lee, 2018; Döring et al., 2018; Kim et al., 2020).

Here, we aimed to establish formate assimilation in cyanobacteria, which use light energy for oxygenic photosynthesis and the CBB cycle for CO_2_ assimilation similar to plants (Hohmann-Marriott and Blankenship, 2011). We selected the model organism *Synechocystis* sp. PCC 6803 (hereafter *Synechocystis*) for the expression of *ftl*, which should enable formate utilization and its conversion into biomass via photorespiratory 2PG metabolism (Figure 1). The study was initiated to test, if formate assimilation can be established in a prokaryotic oxygenic phototroph before making the next step of crop plant engineering. In contrast to our expectations, the knowledge from *E. coli* could be not directly transferred to *Synechocystis*, because instead of improved growth on formate we found marked alteration of cellular C/N metabolism in the *ftl*-expressing strain.

## Materials and Methods

### Strains and Culture Conditions

The cyanobacterial strains used in this work are listed in Supplementary Table S1. The glucose-tolerant strain of *Synechocystis* sp. PCC 6803 served as wild type (WT). Cultivation of mutants and transgenic strains were performed at 50 μg/ml erythromycin (Ery). Axenic cultures of *Synechocystis* were maintained on agar plates (BG 11, pH 8, solidified by 0.9% Kobe agar) at 30°C under continuous illumination of 50 μmol photons m^–2^ s^–1^. The drop-dilution assay was performed with serial dilution of 2 μl cell suspensions at OD_750__nm_ = 1 spotting on agar plates without antibiotics and with different supplements as given in the text. Liquid cultures were grown in the batch mode using BG 11 medium. Cells suspensions were sparked either with ambient air (0.04% CO_2_) or air-enriched with CO_2_ [5% (v/v)] at 30°C under continuous illumination of 100 μmol photons m^–2^ s^–1^. Contamination by heterotrophic bacteria was evaluated by spreading of 0.2 ml of the culture on LB agar plates.

The *E. coli* DH5α cultured in LB medium at 37°C was used for routine DNA manipulations.

### Generation of Transgenic Cyanobacterial Strains

Genes for overexpression and mutation were PCR amplified using gene specific primers (see Supplementary Table S1). All PCR products were ligated with pGEM^®^-T (Promega, Walldorf, Germany) and verified by sequencing (Microsynth Seqlab, Göttingen, Germany). For the overexpression of FTL in *Synechocystis*, *ftl* from *Methylobacterium extorquens* AM1 (Yishai et al., 2017) was amplified with primers adding *Bgl*II and *Mun*I sites at its 5’ and 3’ ends and inserted under control of the strong light-induced P*_psbAII_* promoter into plasmid pAII carrying an erythromycin resistance cassette (Lagarde et al., 2000).

### Protein Extraction From Synechocystis and Western Blot

Twenty ml of *Synechocystis* cells (OD_750__nm_ = 1) were collected by centrifugation at 6000 × *g* for 10 min and immediately frozen in liquid nitrogen and stored at –80°C for further protein extraction. Frozen cells were resuspended in 200 μl homogenization buffer [75 mM Tris−HCl pH 7.5, 1.5 mM EDTA, 1.5 mm PMSF, 1.5 mM NaHSO_3_, 0.15 mM Pefabloc (Merck, Darmstadt, Germany)]. Samples were supplemented with glass beads (diameter 0.5 mm) and subjected to 5 freeze-thaw cycles. Protein quantification was done with Amidoblack (Schulz et al., 1994). The calibration curve was done with different concentration of bovine serum albumin.

SDS−PAGE and Western Blot were done according to standard protocols (Laemmli, 1970; Towbin et al., 1979). The FTL antibody was raised in rabbit against recombinant the generated His-tagged FTL by Davids Biotechnology GmbH (Regensburg, Germany).

### Enzyme Assays

The N-terminal His_6_-tagged *ftl* was obtained after ligation of a *Sac*I/*Kpn*I fragment into pBAD/HisA. The recombinant FTL was purified from cells of *E. coli* strain BL21 (DE3). The pre-cultures were inoculated in fresh LB-medium to an OD_600__nm_ of 0.1 and incubated at 37°C to OD_600__nm_ of 0.6 to 0.8 before induction of *ftl* expression with 0.02% L-arabinose. Expression was carried out for 4 h at 37°C. Cells were harvested by centrifugation at 6000 × *g* for 10 min and washed with lysis buffer [20 mM Tris−HCl pH 7.8, 50 mM NaCl, 10 mM imidazole]. Cells were suspended in lysis buffer supplemented with 1 mg/ml lysozyme and incubated on ice for 30 min. The resulting suspension was subsequently sonicated for 3 × 30 s at maximal power. Lysate was cleared by centrifugation at 14000 × *g* for 30 min at 4°C.

His-tagged proteins were purified via IMAC according to the manufactures protocol (QIAexpressionist, Qiagen) in the gravity flow mode. Lysate passed the Ni-NTA three times, followed by three washing steps with 20 batch volumes washing buffer [20 mM Tris−HCl pH 7.8, 1 M NaCl, 40 mM imidazole]. Elution was done with one batch volume of elution buffer [20 mM sodium phosphate pH 7.8, 500 mM NaCl, 300 mM imidazole] and repeated up to 3 times if desired. Pure recombinant FTL of elution fraction 2 was used for biochemical assays or antibody production.

The FTL activity assay measures the conversion of THF and formate into 10-formyl-THF, which was then quantitatively converted into methenyl-THF by the addition of acid as described (Marx et al., 2003). The assay was performed at 25°C for up to 10 min. Methenyl-THF was determined spectrophotometrically by its characteristic absorption maximum at 350 nm. The 1 ml standard assay mixture contained 0.1 M Tris buffer (pH 8.0), 2 mM tetrahydrofolate (THF) (Merck, Darmstadt, Germany), 10 mM MgCl_2_, 5 mM ATP, 200 mM sodium formate, and 50 μg cell protein extract. The reaction was stopped at different time points (1, 5, and 10 min) by the addition of 2 ml of 0.36 N HCl. The assay was done under low oxygen condition established by a stream of N_2_ to minimize oxidative degradation of the co-substrate THF, whereas the enzyme FTL (EC 6.3.4.3) itself does not contain an oxygen-sensitive cofactor. The absorbance of methenyl-THF was then determined at 350 nm.

Enzyme assay was performed with three technical replicates and given are the mean value ± SD.

### Quantification of Soluble Amino Acids and Organic Acids

Pre-cultures had been cultivated under constant illumination and aerated with 5% CO_2_ in BG11 medium. The cells were diluted to OD_750__nm_ = 1 and shifted to ambient air bubbling either with or without 10 mM sodium formate under constant illumination for 24 h. Free amino acids and organic acids were extracted from frozen *Synechocystis* cell pellets of 10 ml of cultures at OD_750__nm_ = 1 using 80% ethanol at 65°C for 3 h. Cell suspensions were mixed thoroughly by shaking every 30 min. Cell debris were removed by centrifugation at 6000 × *g* for 15 min. the supernatant was lyophilized and re-dissolved in 1 ml MS-grade water (Carl Roth, Karlsruhe, Germany). Amino acids and organic acids were separated through liquid chromatography coupled to tandem mass spectrometry (LC-MS/MS) with Discovery H5 F5 HPLC column (Merck, Darmstadt, Germany) as described in Reinholdt et al. (2019).

All assays were repeated 3 times with independent cell cultivation and three technical replicates each. Pair-wise *t-*test was applied for the statistical comparison of mean values of all 9 data sets.

### RNA-Isolation and Microarray

For transcriptomics, cells were cultured with or without addition of 10 mM sodium formate for 3 days under constant illumination and ambient air bubbling. Cells from 10 ml of cell suspension were harvested by quick centrifugation at 4°C. The cell pellets were frozen in liquid N_2_ and stored at −80°C. RNA isolation, direct RNA labeling and DNA-microarray hybridization were performed as previously described (Gärtner et al., 2019). A high-resolution microarray manufactured by Agilent (Design ID 075764, format 8 × 60 K; slide layout = IS-62976-8-V2) was used for transcriptomic analysis. The array design allows the direct hybridization of total RNA without conversion into cDNA and covers the probes for all annotated genes as well as other transcripts identified in the course of comprehensive RNA sequencing studies. Before labeling, total RNA was incubated with Turbo DNase (Invitrogen) according to the manufacturer’s protocol and precipitated with ethanol/sodium acetate. Further details of the labeling and hybridization protocol can be found in Voß and Hess (2014).

Raw data were further processed with the R package Limma. Median signal intensities were background corrected and quantile normalized. The microarray hybridization was performed with two biological replicates for each treatment. The used array design contained three technical replicates for each single probe and almost all features were covered by several independent probes. Mean values for all probes of a given feature were used for the final calculation of relative transcript ratios normalized to untreated WT. For statistical evaluation, i.e., the *p*-value calculation, the Benjamini–Hochberg procedure was used. Further details of data processing and statistical evaluation using the R software were described previously (Georg et al., 2009). The full array data have been deposited in the GEO database under the accession number GSE143785.

### ^14^C-Formate Uptake

^14^C-Labeled sodium formate was purchased from Merck (Darmstadt, Germany). Pre-cultures had been cultivated under constant illumination and aerated with ambient air and diluted to OD_750__nm_ = 1 prior to the experiment. Given amounts of sodium formate containing 5% w/w ^14^C-labeled sodium formate were added and 1 ml cell suspension were filtered via nitrocellulose membranes (45 μm) and immediately washed with 20 ml BG11 medium at given time points. The membranes were transferred into 5 ml scintillation cocktail (Ultima Gold, PerkinElmer) and analyzed in a scintillation counter (Tri-Carb 2810TS, PerkinElmer). Concentration had been calculated from an individually calibration curve for each experiment. All assays were repeated three times with independent cell cultivation.

### ^13^C-Labeling Pattern Analysis

For stationary isotope tracing of proteinogenic amino acids, cells were pre-cultivated with CO_2_-enriched air (5% CO_2_) in BG11 medium. Cells of the WT or strain exFTL were shifted to ambient air starting with OD_750__nm_ = 0.2 under continuous light and were cultivated for 5 d in the presence of either ^13^C-labeled or unlabeled sodium formate. 2 ml of cells (OD_750__nm_ = 1) were harvested by centrifugation for 5 min at 11 000 × *g*. The pellet was hydrolyzed by incubation with 1 ml of 6N hydrochloric acid for 24 h at 95°C. The acid was evaporated by heating to 95°C. Hydrolyzed amino acids were separated and analyzed as described by Yishai et al. (2017). Hydrolyzed amino acids were separated through ultraperformance liquid chromatography (Acquity, Waters, Milford, MA, United States) using a C_18_-reversed-phase column (Waters) according to previous description. Mass spectra were acquired using an Exactive^TM^ mass spectrometer (Thermo Fisher Scientific). Standards of authentic amino acids (Merck, Darmstadt, Germany) were analyzed under the same conditions in order to determine typical retention times. The program package Xcalibur (Thermo Fisher Scientific) was used for data analysis.

All assays were performed with three biological replicates for each treatment. Representative result shown in here.

## Results and Discussion

### Impact of Externally Supplied Formate on *Synechocystis* WT

**FIGURE 2:**
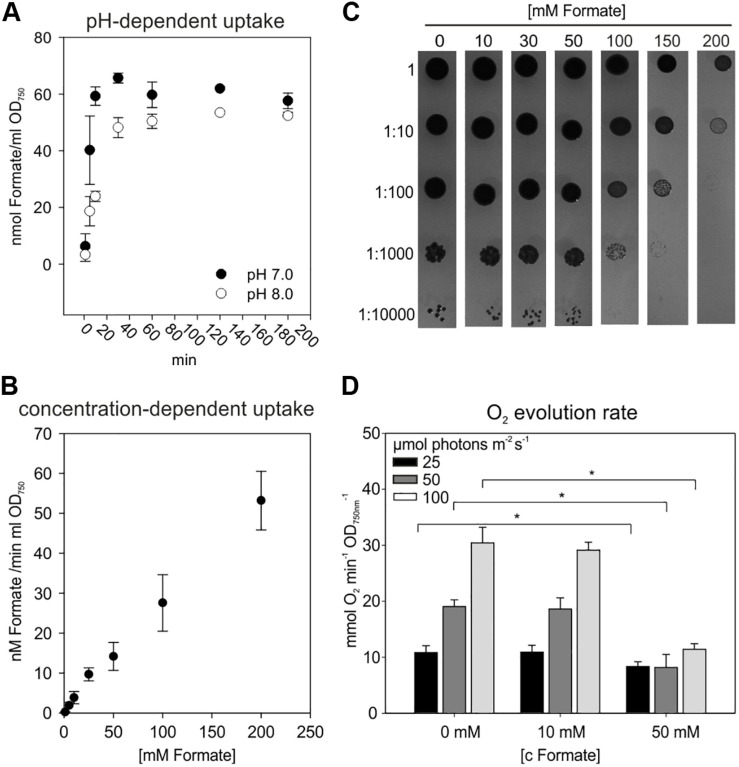
Uptake of sodium formate and its effect on Synechocystis cells. **(A)** Uptake assay using 10 mM sodium formate containing 5% w/w ^14^C-labeled sodium formate with WT cells grown in BG11 at pH 8 and 7, respectively. Given are means of three independent replicates ± SD **(B)** Initial uptake rate of cells incubated for 15 min with 1–200 mM ^14^C-labeled formate at pH 8. Given are means of three independent replicates ± SD **(C)** Tolerance of WT cells toward formate. Serial dilutions of cell suspension (OD_750nm_ = 1) were spotted on BG11 agar plates, pH 8, supplemented with different concentrations of sodium formate (1–200 mM). Pictures show representative results after 7 days under continuous illumination. **(D)** Photosynthetic O_2_ evolution rates in the presence of different concentrations of formate (0–50 mM) and light intensities (25–100 μmol photons m^–2^ s^–1^). Given are means of three independent replicates ± SD (*p < 0.05).

Prior to establish formate assimilation, we tested the ability of *Synechocystis* to import external formate into the cell. To this end, uptake assays using ^14^C-labeled sodium formate were performed to verify formate uptake into *Synechocystis* (Figure 2A). The rapid initial formate accumulation in the cells was followed by saturation after 20^–^30 min. From the uptake measurements we calculated an initial formate uptake rate of 50 nmol formate h^–1^ ml^–1^ OD_750__nm_^–1^ at pH 8. The initial uptake rate increased to 65 nmol formate h^–1^ ml^–1^ OD_750__nm_^–1^, when the assay was performed at pH 7 instead of pH 8. Under more acidic conditions, formate (pKa of 3.74 for formic acid) is less charged what obviously promoted its initial uptake rate. Further analysis of the initial uptake rate of cells supplemented with different concentration of sodium formate revealed a concentration-dependent increase and did not end up in saturation (Figure 2B). Formate, whether dissociated or not, should traverse the outer membrane easily via porins and uptake is rather limited by the permeability of the plasma membrane. Assuming an active transport of the formate anion, a concentration of 200 mM exogenous sodium formate should exceed the affinity of a putative transporter (e.g., FocA in *E. coli* with a K_m_ 119 mM, Wiechert and Beitz, 2017) and end up in no further increase in its uptake rate. However, a linear decrease of the uptake rate correlating with the applied exogenous concentration was observed indicating that non-dissociated sodium formate is entering the cell rather via diffusion than trough specific transporters. Furthermore, a formate-nitrite transporter family, facilitating formate import and export in proteobacteria (Suppmann and Sawers, 1994), was not yet identified in cyanobacteria (Hahn and Schleiff, 2014).

It has been shown that formate can be toxic for oxygenic phototrophs at higher concentrations by interfering with the bicarbonate-binding site at photosystem II (Stemler and Radmer, 1975; Semin et al., 1990; Shevela et al., 2007). Hence, the impact of endogenous formate on *Synechocystis* wild type (WT) was investigated. First, the formate tolerance of WT cells was studied on agar plates supplemented with formate concentrations ranging from 0 to 200 mM (Figure 2C). The growth of *Synechocystis* was unchanged up to 50 mM formate and became somewhat reduced if formate concentrations exceeded 100 mM. Nevertheless, cells survived formate concentrations of up to 200 mM. Second, the growth rates of WT cells were evaluated in liquid media to characterize the long-term impact of formate. No significantly different growth rate was observed in the presence of 10 and 20 mM formate compared to non-treated cells over a time of 7 days. Similar observations were made under different light intensities (50, 100, and 200 μmol photons m^–2^ s^–1^) and different inorganic carbon concentrations (0.04% or 5% CO_2_), respectively (Supplementary Table S2). Third, effects of formate on photosynthesis were studied using *Synechocystis* WT cells exposed to different formate concentrations and light intensities during measuring photosynthetic oxygen evolution. Oxygen evolution was unaffected at 10 mM formate under all tested light intensities (25, 50, or 100 μmol photons m^–2^ s^–1^), but severely inhibited at 50 mM formate at light intensities of 50 and 100 μmol photons m^–2^ s^–1^ (Figure 2D).

Collectively, these data indicated that formate is entering the cells probably via diffusion and low concentrations up to 20 mM of formate were well tolerated by *Synechocystis*, whilst higher concentrations (>50 mM) had a negative impact on photosynthesis and growth. As demonstrated for *E. coli*, the supplementation of 10 mM formate provided sufficient C1 units for serine synthesis via the formate assimilation pathway in a serine auxotrophic strain (Yishai et al., 2017; Kim et al., 2019). Therefore, 10 mM formate was used for subsequent experiments. Generally, the above described experiments verified that *Synechocystis* provides a suitable chassis to implement the formate assimilation pathway.

### Effects of Formate-Tetrahydrofolate Ligase (FTL) Expression on Synechocystis

The heterologous expression of *ftl* should be sufficient to complete a formate assimilation pathway, because all other necessary enzymes are annotated in the *Synechocystis* genome (gene bank accession NC_000911). The FTL from *Methylobacterium extorquens* AM1 was chosen for expression in *Synechocystis*, as the expression of this gene successfully supported formate assimilation in *E. coli* via the desired pathway (Yishai et al., 2018; Kim et al., 2020).

**FIGURE 3:**
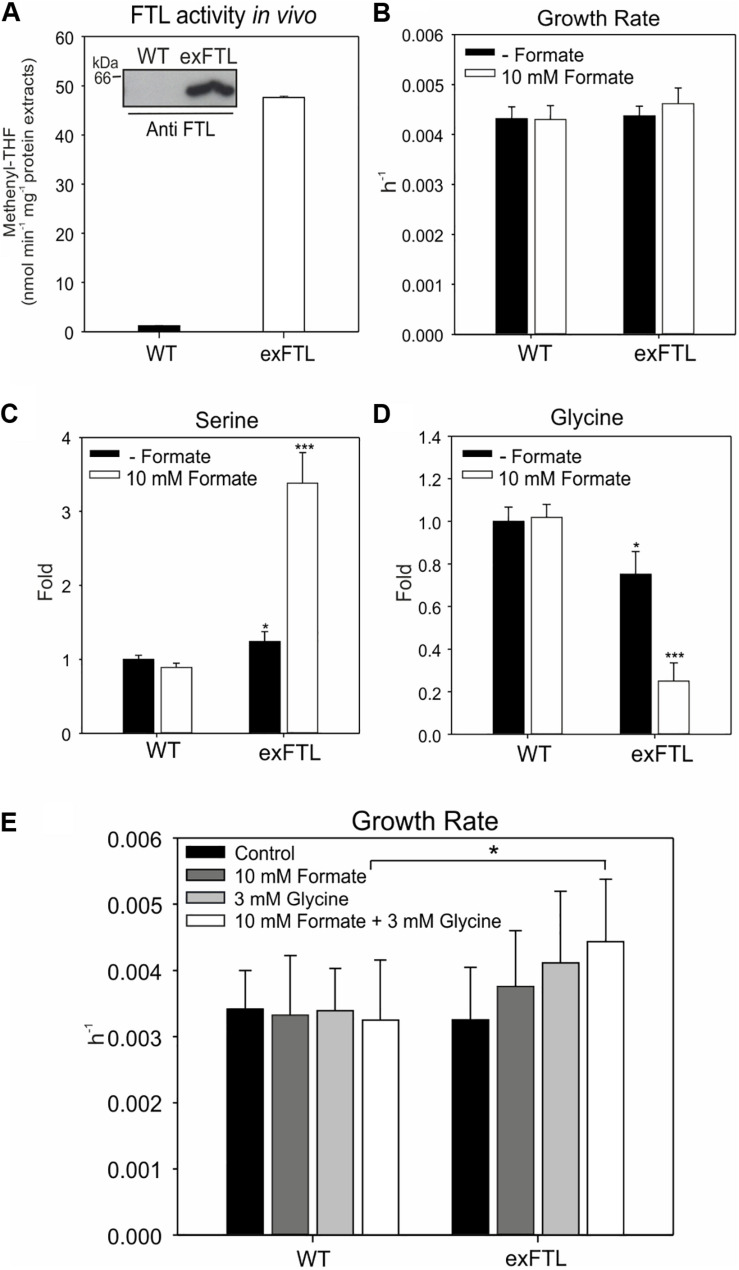
Effect of *ftl* expression and formate supplementation on growth as well as glycine and serine pools in cells of exFTL and *Synechocystis* wild type (WT). **(A)** FTL enzyme activity was compared between exFTL and wild type. Given are mean ± SD from three independent measurements. The inserted figure shows the confirmed expression of FTL in exFTL via immune-decoration with specific antibodies against FTL. **(B)** Growth rates of exFTL and WT incubated with 10 mM sodium formate (white columns) or no formate (black columns). Cells were cultivated at ambient air and 100 μmol photons m^–2^ s^–1^. Given are mean values of three independent cultures ± SD **(C)** Serine and **(D)** glycine levels (expressed as fold changes) in WT and exFTL grown with and without formate supplementation. Cells were cultivated at ambient air and 100 μmol photons m^–2^ s^–1^. Samples were collected 24 h after 10 mM formate addition and analyzed via LC-MS/MS. Given are mean values ± SD of three independent replicates. **(E)** Growth rates of exFTL and WT incubated with sodium formate or/and glycine. Cultures of WT and exFTL were grown and monitored in multi-cultivator at ambient air and 100 μmol photons m^–2^ s^–1^ in BG11 medium supplemented with or without 10 mM formate or (and) 3 mM glycine. 20 mM MgCl_2_ was also added to the medium to alleviate the toxicity of glycine when glycine was supplemented. Given are mean values ± SD of three independent replicates (For all figures: *p < 0.05 and ***p < 0.001 compared to the respective WT sample).

The *ftl* gene was stably inserted into the *psbA2*-site on the *Synechocystis* chromosome, thus, its expression is controlled by the strong, light-induced promoter P_psbA__2_ (Lagarde et al., 2000). Genotype and expression of *ftl* in the resulting exFTL strain were confirmed via PCR, Coomassie-staining, and Western-blotting. Furthermore, FTL enzyme activity was detected in exFTL but not in the WT (Figure 3A). As expected, the expression of *ftl* driven by the *psbA2* promoter resulted in strong protein accumulation in exFTL (Supplementary Figure S1). Phenotyping of exFTL revealed a growth rate similar to WT without additional external formate, while the addition of 10 mM sodium formate led to a minor stimulating effect on exFTL (Figure 3B).

### Metabolic Consequences of *ftl* Expression in *Synechocystis*

**FIGURE 4:**
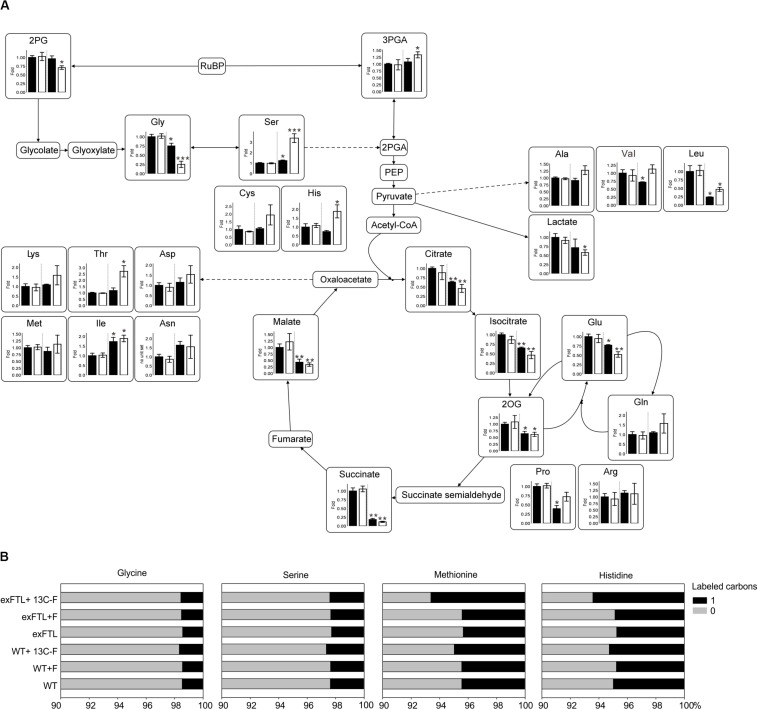
Relative changes of selected metabolites and ^13^C labeling pattern in exFTL compared to *Synechocystis* wild type (WT). **(A)** Cells of WT or exFTL were collected 24 h after addition of 10 mM sodium formate (white columns) or without (black columns) incubated at ambient air and 100 μmol photons m^–2^ s^–1^. Metabolite contents are mean values ± SD relative to the WT mean value from three independent biological replicates.(*p < 0.05; **p < 0.01; and ***p < 0.001 compared to the respective WT sample) 2PG, 2-phosphoglycolate; 3-PGA, 3-phosphoglycerate; Gly, glycine; Ser, serine; Cys, cysteine; His, histidine; Ala, alanine; Val, valine; Leu, leucine; Lys, lysine; Thr, threonine; Asp, aspartate; Asn, asparagine; Met, methionine; Ile, isoleucine; Glu, glutamate; Gln, glutamine; Pro, proline; Arg, arginine; 2OG, 2-oxoglutarate. **(B)** *Synechocystis* WT and exFTL were pre-cultivated under high carbon condition and thereafter transferred to ambient air condition incubated with either unlabeled formate, ^13^C-labeled formate or without formate under ambient air and 100 μmol photons m^–2^ s^–1^ for 5 days. Given are results from one typical experiment.

Next, we compared the metabolome of WT and exFTL in the presence and absence of 10 mM formate. Significant differences, particularly in serine and glycine pools (Figures 3C,D) but also in many other metabolites (Figure 4A) were observed upon formate addition. The addition of formate resulted in approximately 3-fold higher serine levels in exFTL compared to WT, while in the absence of formate only a slight difference was found (Figure 3C). In contrast, glycine decreased approximately 3-fold in exFTL upon formate supplementation, whereas only small differences appeared without formate addition (Figure 3D). The decreased glycine content implied that the amount of glycine, as a precursor of serine in the SHMT reaction, might be the limiting factor for higher formate incorporation into serine. Therefore, growth experiments were performed, in which the medium was supplemented with 3 mM glycine together with 10 mM sodium formate. In addition, 20 mM MgCl_2_ were added to alleviate the toxicity of glycine to *Synechocystis* (Eisenhut et al., 2007). Consistent with our assumption, exFTL showed significantly higher growth rate than WT in the presence of both formate and glycine, whereas only minor, non-significant difference appeared when only glycine or formate was added (Figure 3E).

However, the changes in serine and glycine pools were part of more general metabolic alterations in exFTL, whereas the metabolome of WT was almost unaffected by the addition of formate (Figure 4A and Supplementary Figure S2). For example, the level of the RubisCO carboxylation product 3PGA increased, while the steady state amount of the RubisCO oxygenation product 2PG decreased in exFTL. Furthermore, the expression of *ftl* in *Synechocystis* caused significant decreased contents of 2-oxoglutarate (2OG) and other intermediates of the tricarboxylic acid (TCA) cycle already in the absence of formate. Similar observations were made for the amino acids leucine, proline, histidine, valine and phenylalanine. Some of these changes were intensified by addition of 10 mM sodium formate (Figure 4A and Supplementary Figure S2). In case of valine, the addition of formate rescued the initial decrease to a WT-like level and histidine even exceeded the WT level by 2-fold. For lysine, threonine and asparagine, all originating from oxaloacetate, the contents increased upon formate addition in comparison to non-treated exFTL and WT, respectively.

The two amino acids most directly linked to the C1-pool via their THF-derivatives mediated biosynthesis – methionine and histidine – showed distinct regulations upon formate addition. Whereas the histidine level in exFTL clearly increased upon formate addition, methionine seemed to be unaffected under all growth conditions (Figure 4A and Supplementary Figure S2). Interestingly, the ratio between glutamine and glutamate changed in exFTL upon formate addition (Figure 4A). These changes are usually related to C_i_-limiting conditions and in line with changed 2PG amounts (Eisenhut et al., 2008). Whereas the alterations of other metabolites like citrate, succinate as well as serine and glycine are consistent with a C_i_-limited phenotype, 2PG, 3PGA and 2OG reacted completely oppositional to this hypothesis. Furthermore, not all of these metabolites were affected solely by expression of *ftl* independent of formate addition.

Among all detected metabolites, the alpha aminobutyric acid (AABA) showed the highest relative change. Its levels increased up to 8-fold in exFTL upon formate supplementation (Supplementary Figure S2). This metabolite might originate from serine breakdown to cysteine via oxobutanoate (Supplementary Figure S3), which could also explain the formate-induced increase in cysteine.

To verify whether externally supplied formate was incorporated into cellular biomass, the ^13^C-labeling pattern of proteinogenic amino acids was evaluated in cells grown in the presence of ^13^C-labeled sodium formate for 5 days. The ^13^C-incorporation into the amino acids methionine, histidine, glycine, and serine was analyzed to elucidate whether the C1-building blocks for their biosynthesis derived from ^13^C-formate in exFTL. As expected, glycine was unlabeled (Figure 4B), what proofs that formate oxidation did not occur in *Synechocystis* as it would cause labeling of all amino acids. However, serine also appeared completely unlabeled in exFTL despite its massive accumulation in the presence of formate. Only methionine and histidine were slightly more ^13^C-labeled in exFTL compared to incubation with non-labeled formate (Figure 4B). These results indicated that a rather small amount of formate–derived C1-units was used for methionine and histidine synthesis but not for serine production. Unlike previous studies with designed *E. coli* strains, supplied formate was not the source for the enhanced serine pool in *Synechocystis* (Yishai et al., 2017, 2018; Bang and Lee, 2018; Döring et al., 2018; Kim et al., 2019).

### Analysis of Transcriptome Changes in exFTL

The ^13^C-labeling results revealed that the enhanced serine accumulation in exFTL did not result from significant FTL-mediated formate incorporation. Therefore, the changed serine/glycine ratio likely originated from some regulatory impact of *ftl* expression in *Synechocystis*. To verify this assumption, transcriptomic analyses were preformed using a DNA microarray with RNA isolated from cells of the WT and exFTL, cultured with and without formate. Significant differences in the gene expression between WT and exFTL were detected in the absence of formate, whereas the addition of formate changed the expression pattern in exFTL further (Supplementary Figure S4). In contrast, formate addition had only a minor impact on gene expression in WT cells, which is consistent with the small changes regarding growth and metabolome reported before. Basically, only the *hliB-lilA* (*ssr2595-slr1544*) operon, which encode the small chlorophyll a-binding-like protein ScpD (also called HliB) and LilA that is a member of the extended light-harvesting-like protein family (Kufryk et al., 2008), exceeded the threshold of 2-fold induction. Furthermore, *cmpB* (*slr0041*) and *cmpC (slr0043*) encoding subunits of the ABC-type bicarbonate transporter BCT1 showed more than 2-fold lowered expression (the other genes for the BCT1 transporter were also down-regulated but below significance level, complete data set available under accession number GSE143785).

**FIGURE 5:**
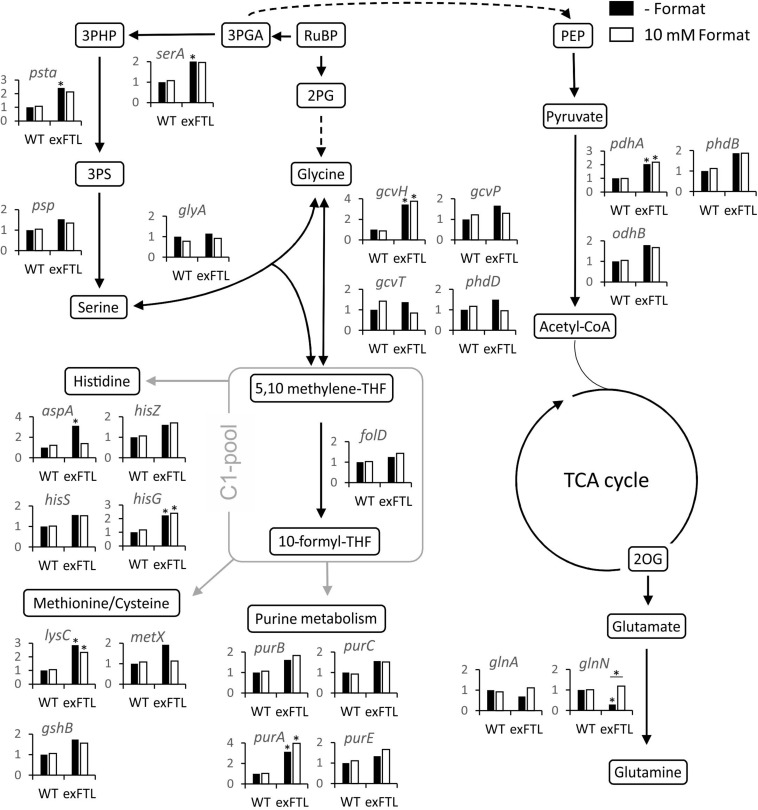
Expression of genes encoding proteins involved in primary carbon and nitrogen metabolism in exFTL compared to *Synechocystis* wild-type (WT). Results are given as relative transcript abundance normalized to untreated WT. The values provided in the figures are means of two biological replicates (*p < 0.05 compared to untreated WT sample) Gene nomenclature is according to KEGG data base.

The global comparison of gene expression revealed that 272 transcripts became significantly (*p* < 0.05) more strongly expressed whereas 232 were more lowly expressed in exFTL compared to WT under both conditions (threshold for significance was 2-fold). The upregulated genes belong to many different categories, comprising many genes for ribosomal proteins and enzymes of the C1, nitrogen and carbon metabolism (for examples see Tables 1, 2). Significant changes in expression were also found for genes involved in serine biosynthesis and related processes, for example photorespiration (Figure 5). To evaluate the FTL-mediated effect on transcript abundance, we initially focused on genes coding for enzymes closely related to FTL activity.

**TABLE 1:** Expression of genes encoding proteins involved in photosynthesis, photorespiration, as well as primary carbon, amino acid and purine metabolism in exFTL compared to wild type (WT).

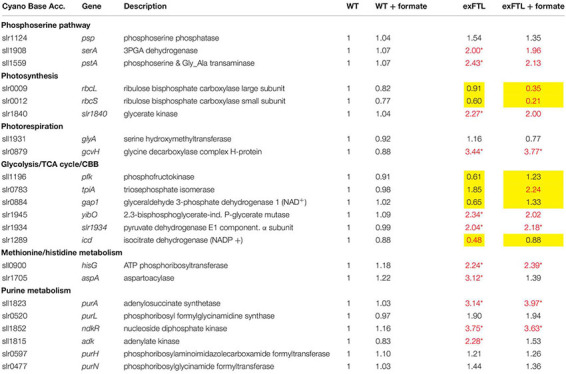

*Synechocystis* cultures of WT and exFTL were cultivated at ambient air supplemented with 10 mM sodium formate or without. Samples from WT and exFTL for RNA isolation were taken after 3 days. Results are given as relative transcript levels normalized to WT without formate addition. Changes that are exceeding the threshold are marked in red. Genes with significant (*p* < 0.05) formate-dependent changes in exFTL are highlighted in yellow (*p < 0.05 to the respective WT sample).

**TABLE 2:** Expression of genes related to folate synthesis and nitrogen metabolism in exFTL compared to wild type (WT).

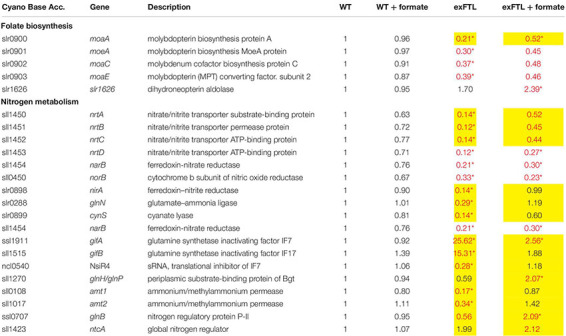

*Synechocystis* cultures of WT and exFTL were cultivated at ambient air supplemented with 10 mM sodium formate or without. Samples from WT and exFTL for RNA isolation were taken after 3 days. Results are given as relative transcript levels normalized to WT without formate addition. Changes that are exceeding the threshold are marked in red. Genes that show strong formate-dependent changes in exFTL are marked in yellow (*p < 0.05 to the respective WT sample).

**FIGURE 6:**
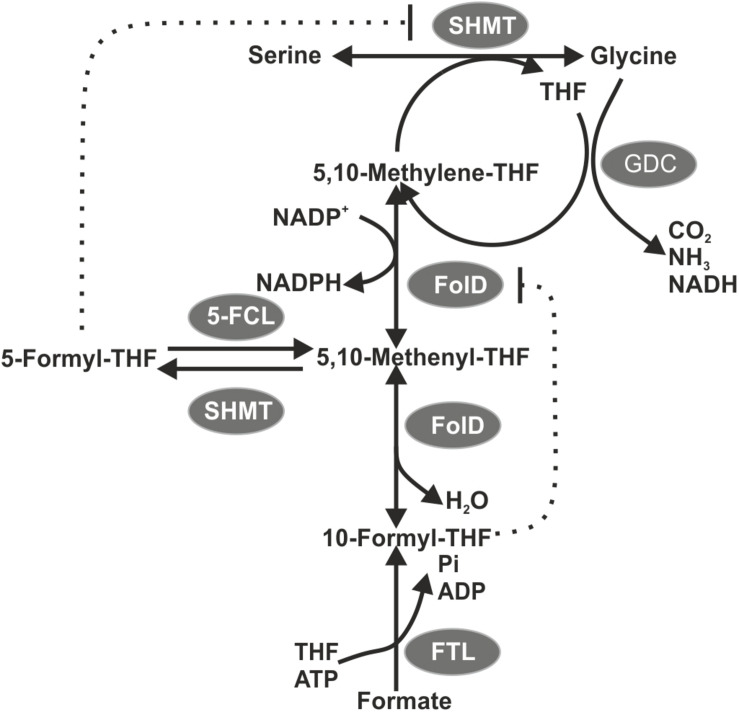
A Scheme displaying the probable interaction of photorespiration and one-carbon (C1) metabolism in *Synechocystis* expressing *ftl*. 5-FCL, 5-formyl-THF cycloligase. Possible inhibitions of serine-hydroxymethyl transferase (SHMT) activity by 5-formyl-THF as well as of 5, 10-methylene- THF dehydrogenase activity by 10-formyl-THF are indicated by dashed lines.

Serine can be synthesized by the photorespiratory 2PG metabolism and by the phosphoserine pathway in *Synechocystis* (Klemke et al., 2015). In the latter pathway serine originates from 3PGA and is made by three enzymatic reactions: 3-phosphoglycerate dehydrogenase (SerA), phosphoserine transaminase (PSTA) and phosphoserine phosphatase (PSP). The genes *serA* and *pstA* were significantly upregulated in exFTL compared to WT, whereas *psp* only showed a slight, non-significant increase in transcript abundance. Upon formate addition expression of none of the three genes was further enhanced, but rather slightly repressed (Figure 5 and Table 1). The serine pool is connected to the glycine pool by the reversible action of SHMT (encoded by *glyA*) that converts serine into glycine and methylene-THF or *vice versa*. Glycine can also derive from the photorespiratory 2PG metabolism, where it is decarboxylated by glycine decarboxylase (GDC). Expression of all four genes for the GDC were coordinately enhanced in exFTL, but only the *gcvH* gene encoding the H protein subunit was significantly up-regulated (genes for P-, T- and L-protein subunits were around 1.5-times higher expressed). However, the addition of formate reduced the expression of genes for the GDC subunits L and P to a WT-like level, which makes it unlikely that the decreased amount of glycine in formate-supplemented exFTL was due to altered GDC expression. Only the expression of *glyA* encoding SHMT was slightly down-regulated upon formate addition. However, expression changes were not consistent with the observed serine accumulation and glycine consumption under formate-supplemented conditions in exFTL, since the strongest transcript changes were observed if this strain was grown under formate-free conditions (Table 1). The slight downregulation of *glyA* expression upon formate addition in exFTL could partially explain the serine accumulation, when we assume that SHMT activity is mostly used to synthetize C1-units by converting serine into glycine and 5,10-methylene THF and to minor extend converts 5,10-methylene-THF and glycine into serine (photorespiratory direction). Furthermore, it is known that SHMT activity is inhibited by 5-formyl-THF (Figure 6), which can be produced from 5,10-methenyl-THF by SHMT itself (Goyer et al., 2005; Collakova et al., 2008). Hence, the combined small downregulation of its expression and possible inhibition of SHMT activity are consistent with the observed serine accumulation upon formate addition in exFTL (Figure 6).

In addition, enzymes involved in purine, methionine and histidine biosynthesis were more highly expressed under formate-free conditions in exFTL than in WT (Table 1), which together with the changed expression of genes involved in folate biosynthesis (Table 2) indicates that the expression of *ftl* interfered with the C1-metabolism of *Synechocystis*. For example, slightly increased expression of the folate-dependent formyltransferases *purN* and *purH* were observed (Table 1). The formyltransferase transfers formyl group from 10-formyl-THF and releases THF during purine biosynthesis, therefore, their increased activity would reduce the 10-formyl-THF pool in exFTL, which could have an impact on other shunts of the C1 metabolism as well. Most probably, the heterologous FTL is changing the balance of the different C1-intermediates bound to THF (see Figure 6) in *Synechocystis*, which is somehow sensed by the cell leading to the observed changes in the transcriptome and metabolome. Furthermore, it has been reported that the dehydrogenase activity of FolD is inhibited by an enhanced 10-formyl-THF pool in *E. coli* FolD (Dev and Harvey, 1978). This FolD inhibition could affect the equilibrium between the C1 units 5,10-methylene-THF and 5,10-methenyl-THF. Furthermore, it was shown with human cell lines that the FolD homolog C-1-tetrahydrofolate synthase is responsible for maintaining the redox balance between NADP^+^ and NADPH (Fan et al., 2014). A similar effect was assumed for a *ftl*-supported Δ*folD E. coli* strain showing a glycine and purine auxotrophy (Sah et al., 2015). Hence, our results indicate that the bifunctional enzyme FolD in *Synechocystis* might be the limiting factor for the assimilation of formate into serine. Most likely, the FTL-related imbalance in the primary and C1 metabolism prevented efficient formate flux into the metabolites of *Synechocystis*.

In contrast to our expectations that FTL expression, especially upon formate addition, mostly influences genes for enzymes associated with C1 metabolism and purine biosynthesis, the strongest changes in transcript abundance were observed for genes connected to nitrogen uptake and assimilation (Table 2). Most pronounced differences were found for the glutamine synthase inactivating factors IF17 and IF7, with a 15-fold and 25-fold enhanced transcript level in exFTL compared to WT (Table 2). In addition, the gene encoding NsiR4, a regulatory sRNA inhibiting translation of IF7 (Klähn et al., 2015) was repressed in exFTL compared to WT, which is consistent with the enhanced mRNA level of IF7. Several other genes that are closely related to nitrogen metabolism in *Synechocystis* (Giner-Lamia et al., 2017) were significantly down regulated in exFTL. For example, this was overserved for the genes encoding the ABC-type nitrate/nitrite transporter (*nrtABCD*), the ammonium/methylammonium permease (*amt1/2*) or the glutamate ammonia ligase (*glnN*) (Table 2). All these genes are regulated by NtcA (*ntcA*: 2-fold enhanced in exFTL, Table 2), the global nitrogen regulator, which senses the 2OG level as a measure of the cellular nitrogen status (Giner-Lamia et al., 2017). 2OG is synthesized in the oxidative branch of the TCA cycle, which is mostly open among cyanobacteria due to the lack of 2OG dehydrogenase complex. Therefore, 2OG is the final product of oxidative degradation of organic carbon and is mainly used as carbon precursor for ammonia assimilation in the glutamine synthase-glutamate synthase (GS/GOGAT) cycle. Hence, 2OG directly links carbon and nitrogen metabolism making it the major signal molecule to sense the C/N ratio *in vivo* (Zhang et al., 2018). The activity of the transcriptional factor NtcA is strongly regulated by the PII protein (*glnB*; 0.6-fold expression in exFTL, Table 2), which in dependence on the amount of 2OG and ATP regulates NtcA activity via an adaptor protein PipX. For example, at high N/C ratios with a lowered 2OG level, PII efficiently binds to PipX and NtcA free of PipX is inactivated, while under low N/C ratios with high 2OG amounts, NtcA-2OG is activated due to strong PipX-binding (Forchhammer and Selim, 2020). According to this model, the decreased 2OG level in exFTL indicates a relative nitrogen-rich status in exFTL (Forchhammer and Lüddecke, 2016). However, formate addition did not change the 2OG level in exFTL but reversed the expression of low-nitrogen-induced genes, for example the genes involved in ammonium uptake and nitrogen assimilation increased to a WT-like level and the transcripts for GS-inactivating factors decreased in comparison to the non-treated exFTL (Figure 3 and Table 2). Hence, the contrary regulation of gene expression and 2OG content in the presence or absence of formate makes a direct impact of 2OG-signaling unlikely, analogous to the C_i_-limited phenotype indicated by the metabolite profile changes described above. In this regard it is interesting to note that *ftl* expression causes a formate-independent downregulation of genes involved molybdopterin synthesis which is slightly stimulated in exFTL upon formate addition (Table 2). Molybdopterin is an essential cofactor for the function of nitrate reductase, the first enzyme initiating the assimilation of the inorganic nitrogen (Berks et al., 1995; Lin and Stewart, 1998). Hence, the reversible regulation of genes for molybdopterin biosynthesis in exFTL with or without formate could impact the nitrate assimilation and explain the strong effects of *ftl* expression on the overall expression of N-related genes.

Compared to genes related to N-assimilation, genes related to carbon fixation (*rbcL, rbcS*) and of the TCA cycle and glycolysis were less affected by the expression of FTL than by the addition of formate (Table 1). The genes encoding RubisCO (*rbcL*, *rbcS*) were significantly lower expressed in exFTL compared to WT in the presence of formate. Some genes for enzymes of the primary carbon metabolism such as triosephosphate isomerase, phosphofructokinase and were stimulated in exFTL upon addition of formate (Table 1). All of them are involved in the breakdown of organic carbon. Therefore, their enhanced expression could be linked to a need for enhanced mobilization of organic carbon reserves due to the lowered RubisCO transcript abundance (e.g., Shinde et al., 2019). The only genes encoding enzymes involved in C metabolism that show an opposite regulation in exFTL upon formate addition were 2,3-diphosphoglycerate-independent phosphoglycerate mutase and the α-subunit of the pyruvate dehydrogenase E1 (Table 2). These findings indicate that formate is probably not used nor sensed as an additional carbon source by *Synechocystis*.

## Conclusion

We aimed to establish a formate assimilation pathway in the cyanobacterium *Synechocystis* to analyze its potential contribution to CO_2_ assimilation via the CBB cycle. Our experiments confirmed that this cyanobacterium represents a suitable chassis for such an attempt, given that external sodium formate is taken up and low amounts of formate are well tolerated without significant effects on growth, photosynthesis, metabolome and transcriptome of WT cells. However, the expression of *ftl* caused defects in sensing the carbon and nitrogen status of the cells. Our results indicate that there is the possibility of an additional, not yet described signal molecule involved in this sensing mechanism. Despite the observed changes in transcript abundances, the exFTL strain grew similarly well as WT under our standard conditions as well as in the presence of formate. These findings show that *Synechocystis*’ physiology can compensate rather large metabolic changes due to transcriptional remodeling without significant effects on growth.

Many other cyanobacteria have been successfully engineered to produce a great variety of biofuels and chemical feedstock, but the observed production titers were usually low (reviewed in Hagemann and Hess, 2018). These limitations could be due to unintended side effects as illustrated here, genetic instability or other yet unknown reasons (Jones, 2014). To minimize these phenomena, comprehensive genetic and metabolic changes of cyanobacteria are necessary as recently shown in the case study on butanol production with *Synechocystis* (Liu et al., 2019). Our study provides an advance toward the possible impacts of metabolic engineering on cyanobacteria.

## Author Contributions

MH conceptualized the project. SS performed the physiological and biochemical experiments. E-MB performed ^14^C-formate uptake experiments and RNA isolation. SL performed analysis of ^13^C-labeled proteinogenic amino acids and data evaluation. ST performed LC/MS-MS measurements. VR and WH performed the microarray analysis and data evaluation. SS, MH, and E-MB made the final data evaluation and wrote the manuscript with contributions from all other authors. All authors contributed to the article and approved the submitted version.

## Conflict of Interest

The authors declare that the research was conducted in the absence of any commercial or financial relationships that could be construed as a potential conflict of interest.
