# Supplementary material for: Expression of Formate-Tetrahydrofolate Ligase Did Not Improve Growth but Interferes With Nitrogen and Carbon Metabolism of Synechocystis sp. PCC 6803
Source: Front Microbiol. 2020 Jul 14;11:1650. doi: 10.3389/fmicb.2020.01650 (PMC7372957; doi:10.3389/fmicb.2020.01650)
Supplement: Supplementary file 1 [file Table_1.DOCX]

**Supplementary Tables:**

**Table S1. Strains and primers used in this work**

| **Strain or primer** | **Genotype or sequence (5’ to 3’)** | **Source or reference** |
| --- | --- | --- |
|  |  |  |
| Wild type | *Synechocystis* sp*.* PCC 6803 | Pasteur culture collection |
| exFTL | PCC 6803 *e*x*ftl* | This work |
| **Primers** |  |  |
| sll1359-fw | AACATATGAAAATAGGAAGAATTACGG | This work |
| sll1359-rev | GGATCCTCAATTCTGGCGTGATCCGG |  |
| ftl-fw | AGATCTATGCATCATCACCATCAC |  |
| ftl-rev | CAATTGTTAGAACAGACCGTCGAT |  |

**Table S2. Growth rates of *Synechocystis* cells under different growth conditions in the presence and absence of formate, respectively.** The growth rates (d^-1^) presented are means ± SE of three independent experiments.

| Growth rate (d^-1^) |  | Light intensity (μmol photons m^-2^ s^-1^) | | |
| --- | --- | --- | --- | --- |
|  | Formate concentration | 50 | 100 | 200 |
| High carbon  (5% CO_2_) | 0 mM | 0.146±0.036 | 0.207±0.043 | 0.244±0.033 |
|  | 10 mM | 0.147±0.018 | 0.183±0.015 | 0.242±0.059 |
|  | 20 mM | 0.147±0.015 | 0.194±0.019 | 0.234±0.048 |
| Low carbon  (air, 0.04% CO_2_) | 0 mM | 0.073±0.018 | 0.122±0.027 | 0.138±0.012 |
|  | 10 mM | 0.084±0.029 | 0.115±0.021 | 0.136±0.006 |
|  | 20 mM | 0.084±0.018 | 0.114±0.025 | 0.14±0.012 |

**Supplementary Figures**

**
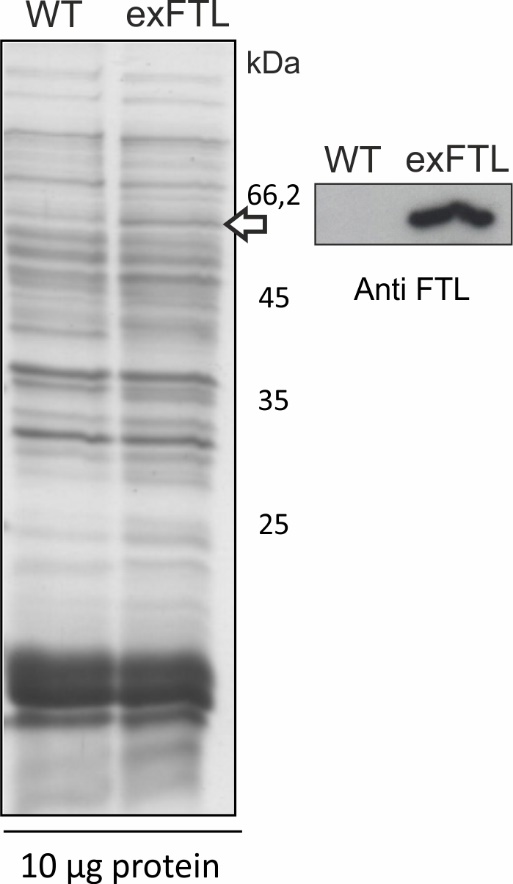
**

**Figure S1: Expression of FTL in *Synechocystis* sp. PCC 6803.**

Expression was confirmed by the appearance of an addition Coomassie stainable band (left, marked with arrow) in total lysate and immune blotting with specific antibodies against FTL (right, see also Fig. 3) in exFTL strain compare to wild type (WT). Size of protein ladder (middle) applicable for SDS-PAGE and Western Blot.

| **Relative folds** | **WT+F / WT** | **exFTL+F / exFTL** | **exFTL / WT** | **exFTL+F / WT+F** |
| --- | --- | --- | --- | --- |
| Asn | 0.96±0.05 | 0.95±0.11 | 1.67±0.38 | 1.63±0.19 |
| Asp | 0.84±0.12 | 1.33±0.22 | 1.15±0.19 | 1.88±0.7 |
| Ser | 0.89±0.05 | 2.74±0.53** | 1.25±0.21* | 3.81±0.65*** |
| Ala | 0.96±0.09 | 1.41±0.14 | 0.91±0.06 | 1.34±0.23 |
| Gly | 1.02±0.22 | 0.34±0.09** | 0.73±0.02* | 0.26±0.13*** |
| Gln | 0.8±0.09 | 1.55±0.29 | 1.11±0.17 | 2.12±0.31 |
| Thr | 0.94±0.02 | 2.35±0.58* | 1.18±0.27 | 2.89±0.61* |
| Met | 1.04±0.06 | 1.3±0.14 | 0.87±0.23 | 1.09±0.36 |
| Cys | 0.97±0.36 | 1.85±0.6 | 1.09±0.29 | 2.24±1.12 |
| Glu | 0.98±0.19 | 0.68±0.14* | 0.78±0.12* | 0.55±0.14** |
| Pro | 1.15±0.08 | 1.84±0.24* | 0.4±0.11* | 0.64±0.16 |
| Lys | 0.81±0.08 | 1.45±0.44 | 1.1±0.14 | 1.95±0.55 |
| His | 1.3±0.25 | 2.54±0.66* | 0.76±0.19 | 1.47±0.32* |
| Arg | 0.8±0.17 | 1±0.44 | 1.16±0.22 | 1.37±0.24 |
| Val | 0.79±0.19 | 1.58±0.21* | 0.72±0.06* | 1.46±0.04 |
| Ile | 1.09±0.38 | 1.1±0.14 | 1.74±0.13* | 1.83±0.29* |
| Leu | 1.05±0.24 | 1.96±0.33 | 0.24±0.04* | 0.45±0.01* |
| Phe | 1.3±0.26 | 1.67±0.24 | 0.54±0.06* | 0.63±0.06* |
| AABA | 0.79±0.1 | 9.12±2.38** | 0.89±0.03 | 10.22±1.72** |
| 2-PG | 1.04±0.17 | 0.83±0.07* | 1.03±0.22 | 0.83±0.09* |
| 3-PGA | 0.97±0.19 | 1.32±0.21* | 1.08±0.12 | 1.48±0.3* |
| 2-OG | 1.08±0.21 | 0.98±0.29 | 0.64±0.04* | 0.59±0.14* |
| Malate | 1.21±0.2 | 0.8±0.12 | 0.43±0.07** | 0.28±0.03** |
| Isocitrate | 0.86±0.1 | 0.71±0.11 | 0.65±0.01** | 0.54±0.08** |
| Lactate | 0.93±0.19 | 0.85±0.16 | 0.71±0.21 | 0.64±0.14* |
| Citrate | 0.86±0.09 | 0.73±0.14 | 0.63±0.01** | 0.53±0.07** |
| Succinate | 1.07±0.18 | 0.64±0.08* | 0.18±0.01** | 0.11±0.02** |


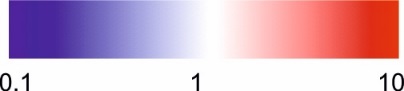


**Figure S2. Heat map showing the relative fold changes of metabolites in exFTL strain compared to wild type (WT).** Samples were collected after cultivation with or without 10 mM formate for 24 h. Cells were cultivated at ambient air and 100 μmol photons m^-2^ s^-1^. Given are mean values and SE of at least three independent replicates. *: *p* < 0.05; **: *p* < 0.01; ***: *p* < 0.001. The color-scale was normalized by Log2 transformation of the relative folds. (+F: 10 mM formate added)


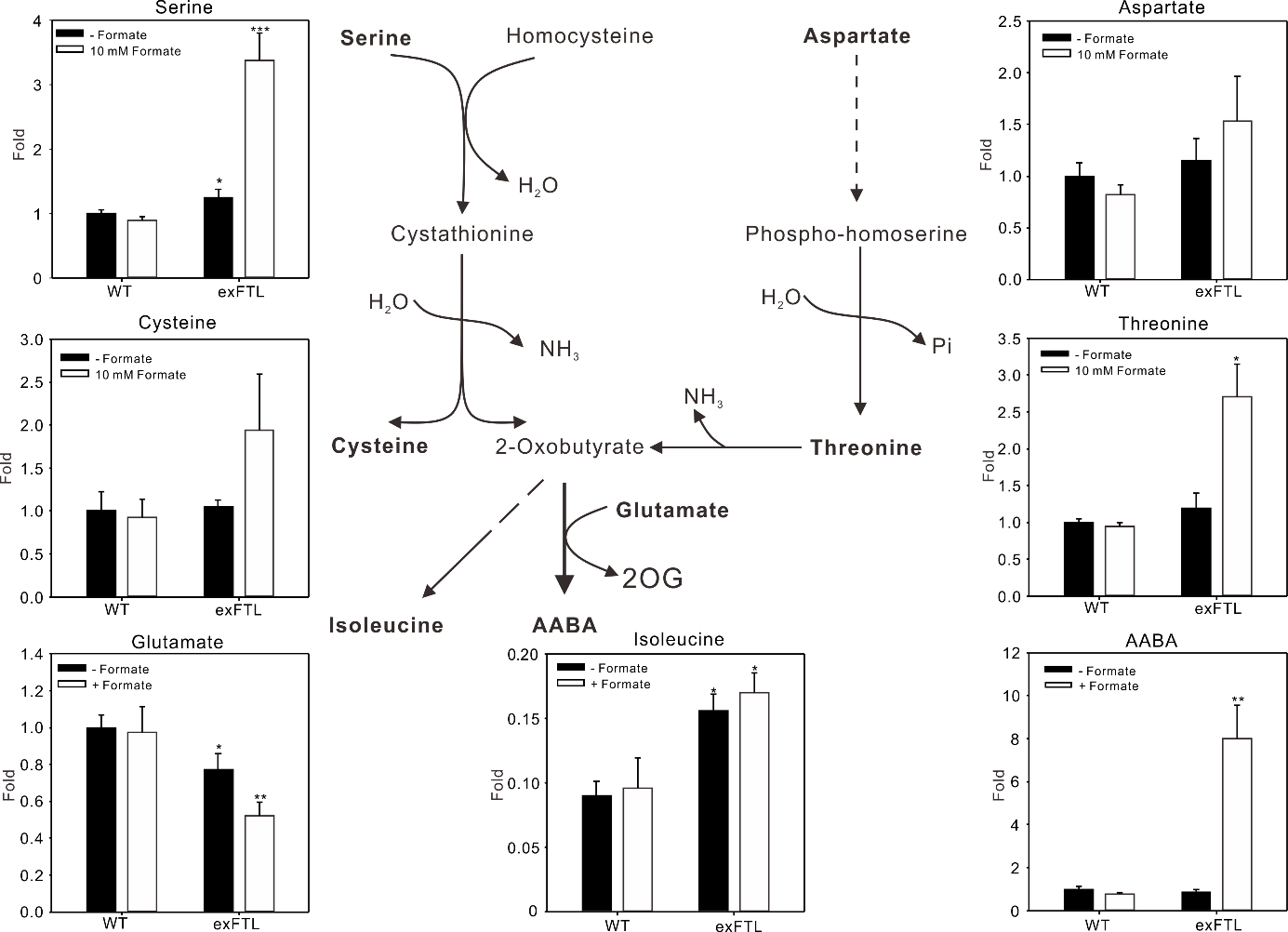


**Figure S3: Changes of metabolites related to alpha aminobutyric acid** **(AABA) metabolism.**

The relative fold changes of metabolites related to AABA metabolism in cells of FTL-expressing strain (exFTL) compared to wild type (WT). Samples were collected after cultivation with or without 10 mM formate for 24 h. Cells were cultivated at ambient air and 100 μmol photons m^-2^ s^-1^. Given are mean values and SE of at least three independent replicates. *: *p* < 0.05; **: *p* < 0.01; ***: *p* < 0.001.


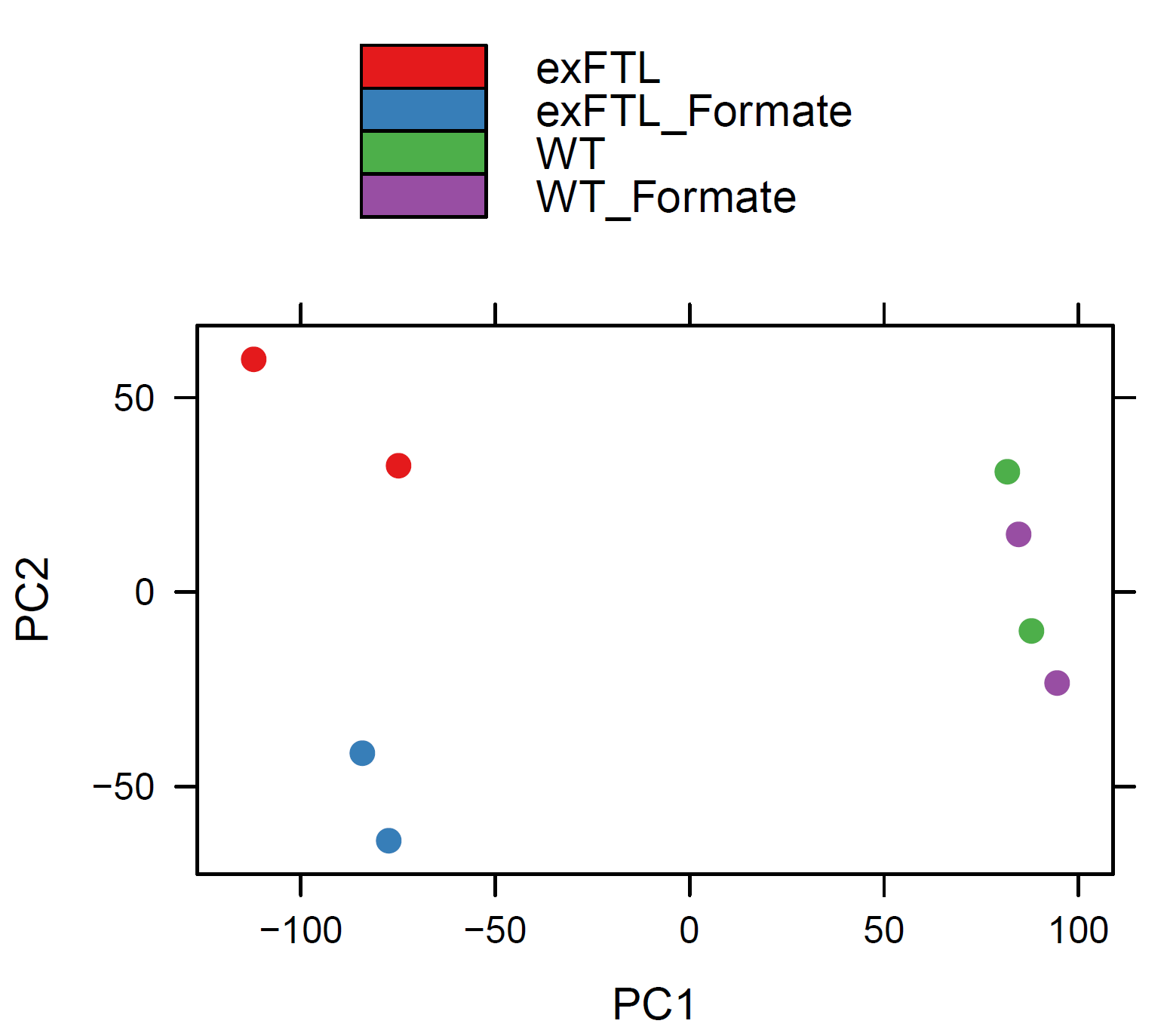


**Figure S4: PCA plot comparing the transcriptomes of WT and exFTL in the presence or absence of formate (10 mM).**
